# High-quality genome of the basidiomycete yeast *Dioszegia hungarica* PDD-24b-2 isolated from cloud water

**DOI:** 10.1093/g3journal/jkac282

**Published:** 2022-10-19

**Authors:** Domitille Jarrige, Sajeet Haridas, Claudine Bleykasten-Grosshans, Muriel Joly, Thierry Nadalig, Martine Sancelme, Stéphane Vuilleumier, Igor V Grigoriev, Pierre Amato, Françoise Bringel

**Affiliations:** Génétique Moléculaire, Génomique, Microbiologie (GMGM), Université de Strasbourg, UMR 7156 CNRS, Strasbourg, France; Lawrence Berkeley National Laboratory, U.S. Department of Energy Joint Genome Institute, Berkeley, CA 94720, USA; Génétique Moléculaire, Génomique, Microbiologie (GMGM), Université de Strasbourg, UMR 7156 CNRS, Strasbourg, France; Université Clermont Auvergne, Clermont Auvergne Institut National Polytechnique (INP), Centre National de la Recherche Scientifique (CNRS), Institut de Chimie de Clermont-Ferrand (ICCF), 63000 Clermont-Ferrand, France; Génétique Moléculaire, Génomique, Microbiologie (GMGM), Université de Strasbourg, UMR 7156 CNRS, Strasbourg, France; Université Clermont Auvergne, Clermont Auvergne Institut National Polytechnique (INP), Centre National de la Recherche Scientifique (CNRS), Institut de Chimie de Clermont-Ferrand (ICCF), 63000 Clermont-Ferrand, France; Génétique Moléculaire, Génomique, Microbiologie (GMGM), Université de Strasbourg, UMR 7156 CNRS, Strasbourg, France; Lawrence Berkeley National Laboratory, U.S. Department of Energy Joint Genome Institute, Berkeley, CA 94720, USA; Department of Plant and Microbial Biology, University of California Berkeley, Berkeley, CA 94720, USA; Université Clermont Auvergne, Clermont Auvergne Institut National Polytechnique (INP), Centre National de la Recherche Scientifique (CNRS), Institut de Chimie de Clermont-Ferrand (ICCF), 63000 Clermont-Ferrand, France; Génétique Moléculaire, Génomique, Microbiologie (GMGM), Université de Strasbourg, UMR 7156 CNRS, Strasbourg, France

**Keywords:** *Tremellaceae*, mitochondrial genome, de novo sequencing, fungi, airborne microorganisms, transposable elements, *Dioszegia hungarica* strain PDD-24b-2, aeromicrobiology, fungal spore, cold environment

## Abstract

The genome of the basidiomycete yeast *Dioszegia hungarica* strain PDD-24b-2 isolated from cloud water at the summit of puy de Dôme (France) was sequenced using a hybrid PacBio and Illumina sequencing strategy. The obtained assembled genome of 20.98 Mb and a GC content of 57% is structured in 16 large-scale contigs ranging from 90 kb to 5.56 Mb, and another 27.2 kb contig representing the complete circular mitochondrial genome. In total, 8,234 proteins were predicted from the genome sequence. The mitochondrial genome shows 16.2% cgu codon usage for arginine but has no canonical cognate tRNA to translate this codon. Detected transposable element (TE)-related sequences account for about 0.63% of the assembled genome. A dataset of 2,068 hand-picked public environmental metagenomes, representing over 20 Tbp of raw reads, was probed for *D. hungarica* related ITS sequences, and revealed worldwide distribution of this species, particularly in aerial habitats. Growth experiments suggested a psychrophilic phenotype and the ability to disperse by producing ballistospores. The high-quality assembled genome obtained for this *D. hungarica* strain will help investigate the behavior and ecological functions of this species in the environment.

## Introduction

There is increasing evidence that airborne microorganisms participate in chemical transformations and physical processes in the atmosphere (Šantl-Temkiv *et al.* 2022). In particular, microorganisms found in clouds play a central role in reactions of carbon-containing compounds at night, whereas during the day, photochemistry is dominant (Vaïtilingom *et al.* 2012, 2013). Both prokaryotic and eukaryotic microorganisms can be found in clouds (Delort *et al.* 2017). Regarding eukaryotes, 1–3% of sequenced 18S rDNA amplicons belong to the class Tremellomycetes of basidiomycete yeasts (Amato *et al.* 2017), which include the genus *Dioszegia* (Order: Tremellales; Family: Tremellaceae/Bulleribasidiaceae) (Liu *et al.* 2015). *Dioszegia hungarica* strain PDD-24b-2 was isolated from cloud water collected at the summit of the puy de Dôme, France (Vaïtilingom *et al.* 2012) (Supplementary Fig. 1) (backward trajectory calculated according to Stein *et al*. 2015). Strains identified as *Dioszegia* sp. are frequently isolated from cloud water sampled at this site (in 70% of studied samples; Vaïtilingom *et al.* 2012). This fungal taxon was repeatedly identified in various cold environments, such as snow and glacial meltwater rivers (De García *et al.* 2007), and is also associated with plants in Antarctica (Ferreira *et al.* 2019). The *D. hungarica* type strain CBS 4214^T^ was isolated from soil in Külsó-tó, Hungary as described in Takashima *et al.* (2001). Also found in warmer environments, it is part of the core fungal community of the wheat phyllosphere (the aerial parts of plants) (Karlsson *et al.* 2017; Sapkota *et al.* 2017). *Dioszegia hungarica* was identified as one of the few “microbial hub taxa” that, when influenced by plant host and abiotic factors, act on the plant microbiome. For example, it directly inhibits the growth of specific bacterial taxa on *Arabidopsis thaliana* seedlings, thus decreasing the phyllosphere bacterial community diversity (Agler *et al.* 2016). The atmospheric environment in which airborne microbes are found represents both a source (immigration) and a sink (emigration) for the phyllosphere microbiome (Kinkel 1997). Examining the genome of *D. hungarica* may provide valuable information to better understand the dynamics of fungal diversity, especially at the plant/atmosphere interface, and its role in climate change-relevant ecosystems (e.g. clouds, cold environments, phyllosphere).

*Dioszegia hungarica*, formerly classified as *Cryptococcus hungaricus* and *Bullera armeniaca* (Takashima *et al.* 2001), is one of the 23 species of *Dioszegia* identified so far (Li *et al.* 2020). To date, genomes of 3 other *Dioszegia* species have been sequenced: *D. aurantiaca* strain JCM 2956 and *D. crocea* strain JCM 2961, isolated from overwintered nettle stems of *Urtica* sp. and strawberry phyllosphere, respectively (Takashima *et al.* 2019), and *D. cryoxerica* strain ANT03-071 (https://mycocosm.jgi.doe.gov/Diocr1), isolated from moss in Antarctica (Connell *et al.* 2010). Previous analyses of the internal transcribed spacer (ITS) and D1/D2 regions of the large subunit rRNA gene showed that *D. hungarica* is phylogenetically distant from these genome-sequenced representatives of the genus (Trochine *et al.* 2017; Li *et al.* 2020). This makes the species *D. hungarica* a good candidate to further investigate fungal genetic diversity. In this study, we describe the high-quality assembled genome sequence of *D. hungarica* strain PDD-24b-2 obtained by a hybrid PacBio and Illumina sequencing strategy. The assembled genome features 17 contigs, 16 large-scale linear contigs and a smaller contig representing the complete circular mitochondrial genome.

## Materials and methods

### Strain and growth conditions

*Dioszegia hungarica* strain PDD-24b-2 was isolated from cloud water collected at the summit of puy de Dôme, France on 17 January 2008 (Vaïtilingom *et al.* 2012). R2A liquid medium was prepared as described previously (Reasoner and Geldreich 1985). Commercial dehydrated R2A agar (Oxoid, Hampshire, UK) was used as solid medium. Yeast mold (YM) medium (pH 6.2) contained per liter 3 g yeast extract, 3 g malt extract, 5 g peptone (pancreatic digest gelatin), 10 g d-glucose, and was supplemented with 20 g agar for solid medium. Liquid cultures were grown at 17°C with agitation (Sanyo MIR 254 refrigerated incubator, MA, USA). The ability to produce ballistospores was assessed on R2A solid medium, placing an inoculated Petri dish above a sterile one as described previously (Ianiri *et al.* 2014).

### DNA extraction and PCR amplification

Total DNA was extracted from a 4-day aerobic culture (OD at 600 nm of 0.97) in 200 mL R2A medium incubated at 17°C, using the MasterPure complete DNA and RNA purification kit as described by the manufacturer (Lucigen, WI, USA). The 18S rRNA gene was PCR-amplified from total DNA (25 ng) using primers Dios20F (5′-GTGCGTCTGATTCTTGACTCC-3′) and Dios11R (5′-CCCGACCGTCCCTATTAATCA-3′) and DreamTaq DNA polymerase, as recommended by the manufacturer (Thermo Fisher Scientific Baltics, Vilnius, Lithuania). The PCR program (Biometra TOne thermocycler, Analytik Jena, Jena, Germany) involved DNA denaturation at 95°C for 5 min, 30 cycles of 45 s at 93°C, 20 s at 56°C and 1 min at 72°C, and a final 10 min extension at 72°C. The amplified 1,080 bp PCR fragment was sequenced by the Sanger method (Microsynth France, Vaulx-en-Velin, France).

### Genome sequencing, assembly, and automatic annotation

Illumina library preparation (Nextera XT kit), PacBio library preparation (SMRTbell express template prep kit 2.0) and high throughput sequencing of *D. hungarica* PDD-24b-2 were performed by GenoScreen (Lille, France). Libraries were sequenced using the MiSeq Illumina platform and the PacBio platform (SMRT cell Pacbio Sequel). Illumina and PacBio reads were quality checked with FastQC v0.11.9 (Andrews, Braham Bioinformatics).

Illumina adapter sequences were removed with CutAdapt v2.10 (Martin 2011) and paired-end reads cleaned with Prinseq v0.20.4 (Schmieder and Edwards 2011): the first 15 nucleotides of each read were cut, nucleotides with a Phred score under 30 were cut from the read 3′ end, reads shorter than 60 nucleotides were discarded, reads with a mean Phred score under 30 were discarded, as well as those containing undetermined nucleotides. Only paired reads were conserved. After these processing steps 8,383,275 read pairs were obtained.

PacBio subreads were assembled with Flye v2.8.2 (Kolmogorov *et al.* 2019). The cleaned Illumina read pairs were used to correct the PacBio assembly using BOWTIE2 v2.4.1 (Langmead and Salzberg 2012; Langmead *et al.* 2019) and Pilon v1.23 (Walker *et al.* 2014). Contigs were aligned to each other using BLASTn v2.10.1 (Camacho *et al.* 2009) to resolve alternative haplotypes. Telomeric repeats of the sequence T_2_AG_3–5_, akin to those of *Cryptococcus neoformans* (Edman 1992) were searched and visualized in this assembly using IGV v 2.12.0 (Robinson *et al.* 2011). Completeness of the *D. hungarica* PDD-24b-2 genome assembly was assessed with BUSCO v5.2.2 (Manni *et al.* 2021) against tremellomycetes_odb10, and compared with the 3 previously released genomes for the *Dioszegia* genus.

The nuclear genome was deposited in the MycoCosm platform (Grigoriev *et al.* 2014) and automatically annotated, as previously described (Kuo *et al.* 2014) using the JGI Annotation Pipeline. The mitochondrial genome annotation pipeline combined ab initio predictions, homology-based predictions with a curated mitochondrial protein set, and Hidden Markov models (HMM) based predictions, as described in Haridas *et al.* (2018). The EuKaryotic Orthologous Groups (KOG) classification scheme was used to evaluate the number of genes associated with predicted processes with detailed gene ID available at https://mycocosm.jgi.doe.gov/cgi-bin/kogBrowser?type=KOG&db=Diohu1. The Kyoto Encyclopedia of Genes and Genomes (KEGG) pathway database was used to identify metabolic pathway genes (https://mycocosm.jgi.doe.gov/cgi-bin/metapathways?db=Diohu1).

### Identification of TEs

Putative TEs were searched in the *D. hungarica* PDD-24b-2 genome sequence using 2 de novo approaches: the RepeatModeler v2.0.3 pipeline (Flynn *et al.* 2020) with its long-terminal repeat (LTR) pipeline extensions, and the Extensive de novo TE Annotator pipeline (Bell *et al.* 2022). TEs were also identified by protein homology with transposon sequences from other fungi (*Saitozyma podzolica*, *Cryptococcus neoformans*, *Cryptococcus gattii*, *Rhodotorula toruloides*, *Candida glabrata*) using BLAST + tblastx v2.11.0 (Camacho *et al.* 2009). Detected sequences were manually curated using CD Search (Marchler-Bauer and Bryant 2004; Marchler-Bauer *et al.* 2017) to predict conserved protein domains. Target site duplications (TSD) were identified by manually checking for direct repeats in sequences adjacent to identified TEs, and confirmed by surveying several copies. Detected putative TEs were classified into Orders and Superfamilies, as previously described (Wicker *et al.* 2007). Unique candidate TEs for each family, with typical TE domains (e.g. transposase, reverse transcriptase, RNase H, integrase, aspartic protease, *gag* domains) were then used to build a putative TE library (Supplementary File 1) to screen the genome sequence of *D. hungarica* PDD-24b-2 using RepeatMasker v4.1.2-p1 and estimate putative TE copy number, including full-length and truncated copies. The RepeatMasker.out file was parsed with the tool “One code to find them all” (Bailly-Bechet *et al.* 2014) to assemble detected TE fragments. More information on the TE-mining process can be found in the following GitHub repository: https://github.com/JarrigeD/Dioszegia_hungarica_sequencing.

### Phylogenetic analysis

The ITS region (ITS1, 5.8S ribosomal RNA gene, ITS2 and large subunit ribosomal RNA gene partial sequence) phylogenetic tree of the *Dioszegia* genus was constructed entirely with MEGA11 (Tamura *et al.* 2021). An alignment of the ITS region was generated using MUSCLE (Edgar 2004) on a total of 462 positions for *D. hungarica* PDD-24b-2, reference strains of the 23 *Dioszegia* species described to date, and 2 *Hannaella* type strains used as outgroup (Supplementary Table 2). The MEGA11 “find best fit substitution model” tool was used to choose the substitution model for tree building. The General Time Reversible model with rate heterogeneity across sites (GTR + G) (Tavaré 1986; Yang 1996) had the lowest Bayesian information criterion score and corrected Akaike information criterion score and was used to calculate the matrix of pairwise distances. A discrete Gamma distribution was used to model evolutionary rate differences among sites [5 categories (+G, parameter = 0.1890)]. A total of 500 replicate trees were built with the Maximum Likelihood method to calculate bootstrap support values, and the best tree topology with the highest log likelihood (−2379.73) was selected.

### Geographical distribution

A total of 2,068 whole-genome shotgun (WGS) raw read metagenomic datasets were hand-picked to maximize geographic and environmental variety and retrieved from the Sequence Read Archive (SRA) using sra-tools. The presence of *D. hungarica* sequences was tested using sra-tools blastn_vdb megablast, with strain PDD-24-2b-2 ITS region as query and a minimum percentage identity threshold of 97%. The resulting BLAST hits were filtered to target members of the genus *Dioszegia* (≥45 nt with ≥99% identity to the PDD-24b-2 5.8S rRNA gene sequence) and of the species *D. hungarica* (≥15 nt to ITS1 or ITS2 sequences, E-value ≤10e^−10^). These thresholds were defined using alignments of ITS regions of *D. hungarica* PDD-24b-2 to those of fungal type strains in the NCBI ITS_RefSeq_Fungi database. Maps of WGS datasets with *D. hungarica* or *Dioszegia* sp. hits were plotted in Python v3.10.2 using Matplotlib v3.5.1 and GeoPandas v0.10.2. Details of metadata and dataset accessions, homolog search scripts, filtering parameters, and mapping processes are available at https://github.com/JarrigeD/Dioszegia_hungarica_sequencing.

The GlobalFungi database (accessible at https://globalfungi.com/, last accessed on 2022-09-12) (17,000 ITS amplicons environmental samples) (Větrovský *et al.* 2020), the MarDB database (accessible at https://mmp.sfb.uit.no/blast/, last accessed on 2021-11-23) (14,600 marine microbial genomes) (Klemetsen *et al.* 2018; Priyam *et al.* 2019) and TARA Ocean Gene Atlas databases (Villar *et al.* 2018) (accessible at https://tara-oceans.mio.osupytheas.fr/ocean-gene-atlas, last accessed on 2022-09-12) EUK_SMAGs (713 eukaryotic plankton Metagenome Assembled Genomes: MAGs) (Delmont *et al.* 2022), MATOUv1_metaG (116.8 million eukaryotic expressed genes + 530 Arctic Ocean MAGs) (Carradec *et al.* 2018), and OM-RGC_v2_metaG (370 marine metagenomes + 530 MAGs) (Salazar *et al.* 2019) were also searched for close homologs of the ITS region of *Dioszegia hungarica* PDD-24b-2.

## Results and discussion

### Cell morphology and growth characteristics

**Fig. 1.**
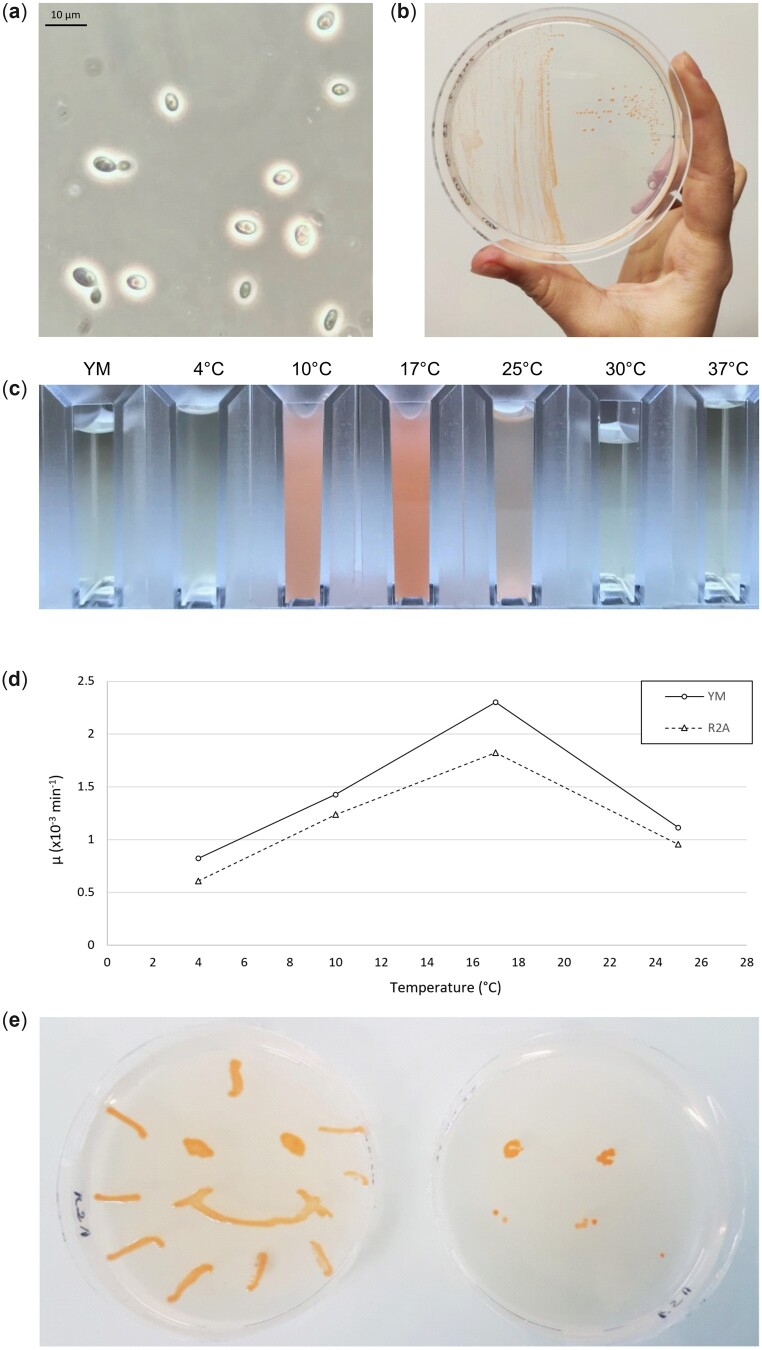
Morphological and growth characteristics of *D. hungarica* strain PDD-24b-2. a) Cellular morphology observed with a Leica DM4000 B microscope at ×1,000 magnification after growth in YM broth at 17°C. b) Colonies on R2A solid medium at 17°C. c) Liquid cultures after growth at different temperatures for 4 days. Highest cell density was observed at 17°C. No growth was observed above 25°C or in sterile YM medium. d) Effect of culture incubation temperatures on growth rate (µ). For each medium (YM or R2A), the mean growth rate was the mean of 2 biological replicates. e) Ballistospore production on solid R2A medium. The inoculated plate (left) was placed on top of the uninoculated one (right). After 6 days of incubation, colonies also appeared on the bottom plate as a partial mirror image of the top inoculated plate.

Single ovoid cells of ∼4 µm in length, dividing by polar budding, were observed by optical microscopy (Fig. 1a). *Dioszegia hungarica* strain PDD-24b-2 grows in R2A and YM media with the characteristic deep orange color typical of this genus (Inacio *et al.* 2005), which becomes more pronounced at higher cell density (Fig. 1b and c). Growth in YM was faster than in R2A (Fig. 1d), with incubation temperature strongly affecting growth (Fig. 1d). The shortest doubling times were observed at 17°C in both YM (297 ± 5 min) and R2A (381 ± 33 min) media. Incubation at 4°C and 25°C resulted in 3- and 2-times longer doubling times, respectively. No growth was observed at 30°C and 37°C after 2 weeks under all tested conditions (data not shown). This is in line with the temperatures of low-altitude clouds at the French site from which the strain was isolated, i.e. 5°C mean and 17°C maximal temperature, respectively (Vaïtilingom *et al.* 2010). In addition, strain PDD-24b-2 was able to produce ballistospores, a launched spore type specific to basidiomycetes, at 17°C on R2A solid medium after 6 days of culture. Ballistospores are able to launch from an inoculated plate to a neighboring sterile one, on which colonies will grow following incubation, forming a “mirror” image of the inoculated plate (Fig. 1e). Ballistosporic basidiospores have been proposed to act as giant cloud condensation nuclei that could increase precipitation by coalescing smaller droplets (Hassett *et al.* 2015). Unlike the strain studied in this work, however, one of the *D. hungarica* strains isolated from terrestrial habitats was unable to produce ballistospores (Takashima *et al.* 2001), suggesting that this trait is not conserved within *D. hungarica*.

### Genome sequencing, assembly, and completeness

The genome of *D. hungarica* PDD-24b-2 was sequenced by a hybrid strategy using a combination of PacBio (average coverage of 97×, median subread size of 3,584 bp, and 474,621 subreads in total) and Illumina (average coverage of 101×; 9,901,968 read pairs of 151 bp) sequencing, yielding a high-quality assembly. The 28 contigs assembled from PacBio subreads were corrected with the Illumina pair-end reads. BLASTn alignment analysis identified 2 identical contigs which were merged, and 9 small contigs nearly identical to larger ones (between 99.96% and 100% identity) which could represent alternative haplotypes and were thus discarded from the final assembly. This yielded a final genome assembly of 18 contigs, with 17 linear contigs corresponding to the nuclear genome. One contig was circular, as evidenced by more than 500 Illumina reads bridging its ends (data not shown) and corresponded to the mitochondrial genome. Its size of 27,226 bp was in close agreement with that estimated for the *D. hungarica* strain CBS 4214^T^ from average contour-length on electron micrographs 20 years ago (27.3 kb, Gácser *et al.* 2002).

**Fig. 2.**
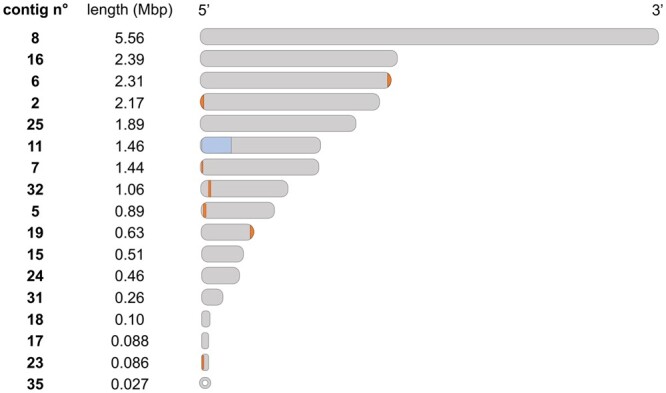
Telomeric sequences distribution in assembled contigs of *D. hungarica* PDD-24b-2. Detected telomeric T_2_AG_3–5_ repeats are indicated as dark bars (not drawn to scale). The complete ribosomal RNA gene region (with an estimated 35 copies of the ribosomal RNA gene cluster) is highlighted on contig11. Identification labels for assembled contigs are given as in MycoCosm (https://mycocosm.jgi.doe.gov/Diohu1). Contig 35 corresponds to the circular mitochondrial genome.

After assembly, the beginning 5′ third and the remaining 3′ parts of the 18S rRNA gene were located at the termini of 2 nuclear contigs, i.e. the smallest contig of 2 kb (contig20) and the contig of 1.11 Mbp (contig11, which also contained the remainder of the rRNA-encoding region). To confirm the linkage between contig20 and contig11, PCR amplification of the 18S rRNA gene was performed using primers targeting contig20 and contig11. The full 18S rRNA gene sequence including the 19 nt gap initially left out of the assembly was sequenced. Accordingly, contig11 was merged with the smaller contig20 to restore a complete 18S rRNA gene within the reunited rRNA region composed of 5S rRNA, 18S rRNA, 5.8S rRNA, and 25S rRNA genes. Genome regions with rRNA genes are notoriously difficult to resolve in eukaryotic genomic assemblies, as rRNA genes can be found in tens to thousands tandem copies (Nelson *et al.* 2019). For instance, *Cryptococcus neoformans*, another basidiomycetous yeast, contains around 55 tandem repeats of a single rRNA gene region (Loftus *et al.* 2005; Ganley and Kobayashi 2007). The rRNA gene copy number is usually estimated by relative read coverage (Lofgren *et al.* 2019). For strain PDD-24b-2, Illumina read depth coverage of contig20 was 35 times higher than for the rest of the genome (Supplementary Fig. 2). As expected, a similar increased coverage was also observed for the terminal part of contig11 in which the rRNA gene cluster is located. This suggests that ribosomal RNA genes are present in about 35 copies in *D. hungarica* PDD-24b-2, although the precise number of tandem repeats remains unknown. To estimate the length of the whole region containing copies of the rRNA gene cluster, we multiplied the rRNA unit length (10.29 kb) by its relative coverage of 35. The estimated length of the complete contig11 would thus be ∼1.46 Mbp (Fig. 2).

For 7 contigs, T_2_AG_3-5_ telomeric repeats were detected at one of the ends only (Fig. 2), suggesting incomplete resolution of the nuclear genome. Nevertheless, the statistics and characteristics of the obtained final genome assembly of *D. hungarica* strain PDD-24b-2, with 16 large-scale contigs and a complete mitochondrial genome contig, compare favorably with previously reported genomes for the genus *Dioszegia* (Table 1). Specifically, and with an L50 value of 4 and a N50 length of 2.17 Mbp, the genome assembly of *D. hungarica* PDD-24b-2 contains no gaps, unlike the 3 previously sequenced genomes of *Dioszegia* strains (Table 1). In particular, the assembly of the *D. cryoxerica* ANT 3-071^T^ genome was strongly fragmented, with 111 scaffolds under 2 kb, a L50 of 96 and a N50 of 0.12 Mbp. In the case of *D. aurantiaca* JCM 2956^T^ and *D. crocea* JCM 2961^T^, some large and a few small (<2 kb) scaffolds were reported.

**Table 1.** Genome assembly and predicted annotation data of *D. hungarica* PDD-24b-2 compared with the 3 other genome-sequenced *Dioszegia* sp. Annotation statisticsBUSCO v5.2.2 assembly completeness assessment (%)^c^Complete

Content	*Dioszegia hungarica* PDD-24b-2	*Dioszegia aurantiaca* JCM 2956^T^	*Dioszegia crocea* JCM 2961^T^	*Dioszegia cryoxerica* ANT 03-071^T^
BioProject accession no.	PRJNA809585	PRJDB3721	PRJDB3718	PRJNA196046
Reference	This study	Takashima *et al.* (2019)	Takashima *et al.* (2019)	L.B. Connell (personal communication)
Sequencing and assembly^a^ statistics				
Sequencing read coverage depth (technology)	97× (PacBio Sequel) + 101× (Illumina MiSeq)	112× (Illumina HiSeq 2500)	176× (Illumina HiSeq 2500)	98.5× (Illumina HiSeq 2500)
Assembly size (Mbp)	20.96	19.34	20.60	39.52
Scaffolds/contigs	18/18	52/139	26/86	865/1,318
Longest scaffold (Mbp)	5.56	4.12	3.59	0.43
L50 scaffold value	4	5	10	96
N50 scaffold length (Mbp)	2.17	1.28	1.95	0.12
Scaffolds over 2 kb	17^b^	44	21	754
GC content (%)	57.2	53.6	53.2	56.9
Gaps (%)	0.00	0.82	0.59	1.3
Linear contigs	16^b^	139	86	1.318
Mitochondrial genome (kb)	27 (circular)	NA	NA	36
				
Gene models	8,219	8,106	8,753	15,948
Average transcript length (bp)	1,538	1,817	1,801	1,415
Average exon/intron length (bp)	247/67	258/59	259/61	264/61
Average exons per gene	6.23	5.90	5.80	5.36
Average protein length (aa)	513	507	500	429
Genes with GO annotations	3,925	NA	NA	6,951
				
89.3	89.0	89.3	90.0	
Single	88.8	88.9	88.7	36.6
Duplicated	0.5	0.1	0.6	53.4
Fragmented	3.3	3.9	3.9	3.2
Missing	7.4	7.1	6.8	6.8

a Haploid assemblies except for *D. cryoxerica* ANT 03-071^T^ which is probably diploid.

b Scaffolds and linear contigs left after merging contig20 and contig11.

c BUSCO reference dataset: tremellomycetes_odb10 (2021 June 28).

NA, not available.

The GC content of *D. hungarica* is about 57%, similar to that reported for *D. cryoxerica*, and higher than about 53% for *D. aurantiaca* and *D. crocea.* A detailed comparative assembly assessment of the 4 genomes using BUSCO v5.2.2 (Manni *et al.* 2021) was performed using the tremellomycetes_odb10 database. The estimated occurrence of complete genes was similar for the 4 compared *Dioszegia* genomes, yet at about 89.5% instead of the expected 100%. This suggests a lineage specific bias for the *Dioszegia* genus of the reference tremellomycetes_odb10 database. A notable difference between *Dioszegia* genomes is the high percentage of duplicated genes reported for *D. cryoxerica* (53.4%), possibly reflecting unresolved haplotypes in the diploid assembly of its genome (https://mycocosm.jgi.doe.gov/Diocr1). This would also be consistent with the twice larger length of its assembly (39.5 Mpb) compared with the 3 other reported genomes including *D. hungarica*.

### Genome annotation for protein-coding genes and predicted metabolic pathways

The obtained number of 8,219 predicted protein-coding genes is close to that reported for *D. aurantiaca* JCM 2956^T^ and *D. crocea* JCM 2961^T^ (Table 1) and also to the total number of unique protein-coding genes of *D. cryoxerica* ANT 3-071^T^. The KOG classification scheme was used to evaluate the number of genes involved in cellular processes and signaling (1,301), information storage and processing (1,067), metabolism (1,460), and genes with unknown functions (1,056) (detailed gene ID available at https://mycocosm.jgi.doe.gov/cgi-bin/kogBrowser?type=KOG&db=Diohu1). The largest gene families include transporters from the Major Facilitator Superfamily (138) and sugar transporters (66), protein kinases (102), and clusters of genes with WD domain (100) and helicase-domain (75). Secondary metabolism is represented by 3 NRPS-like gene clusters and a single PKS-like gene cluster.

Gene predictions were analyzed in the light of experimentally characterized metabolic traits in *D. hungarica* (Takashima *et al.* 2001). Genes for glycolysis/gluconeogenesis (39 genes), the TCA cycle (19 genes), starch utilization and production (61 genes), and nitrite utilization (1 nitrite reductase-encoding gene) (KOG classification within the MycoCosm plateform) reflect the previously reported utilization by *D. hungarica* of glucose, succinic and citric acid, starch, and nitrite, respectively. Conversely, no genes were predicted for methanol or nitrate utilization, or for thiamine biosynthesis, confirming the reported inability of *D. hungarica* to use methanol or nitrate, and its thiamine auxotrophy. Identified genes for carotenoid biosynthesis (36 putative genes, KEGG annotations, JGI Annotation Pipeline) are in line with previous reports of carotenoids in *Dioszegia* strains (Madhour *et al.* 2005; Amaretti *et al.* 2014; Villarreal *et al.* 2016) and also with the bright orange color culture observed for *D. hungarica* PDD-24b-2 (Fig. 1). Carotenoids prevent oxidative stress (Madhour *et al.* 2005) and act as photo-protectants (Moliné *et al.* 2009) and cryo-protectants (Dieser *et al.* 2010), and may thus favor survival under the harsh conditions of clouds (Šantl-Temkiv *et al.* 2022). In this context, strain PDD-24b-2 also encodes a putative antifreeze protein (protein ID: 32937), with a predicted ice-binding protein domain (InterPro entry: IPR021884) and a predicted secretion signal. Secreted antifreeze proteins impair ice crystal formation and protect cell integrity under cold conditions (Hashim *et al.* 2013), suggesting a role of this protein in cold protection of *D. hungarica* in the cloud environment that remains to be experimentally validated.

### Transposable elements

A total of 311 putative sequences related to TEs were detected and classified in 16 TE families (Supplementary Table 1 and File 1). TEs are dominated by Class I elements representing 12 families. Of those, 7 families of *Copia* and 1 family of *Gypsy* LTR TE were found. Class I non-LTR elements putative families were distributed in 3 LINE families and one DIRS family. Four families of Class II terminal inverted repeat (TIR) elements were also detected. Only 1 family encodes a transposase gene carrying a cl24015 domain attributed to MULE TE DDE transposases (Babu *et al.* 2006). The 10 bp long TSD supports an assignation to the *Mutator* Superfamily. Four families of nonautonomous Miniature Inverted-Repeat Transposable Elements (MITE) were also detected. One of them is related to the aforementioned *Mutator* element (same TIR and 10 bp long TSD). The others may be related to the hAT superfamily, according to their TSD length of 8 bp. However, we could not detect the corresponding autonomous copies encoding the transposases to confirm their annotation. In total, putative transposon-related sequences (around 130 kb) represent 0.63% of the *D. hungarica* PDD-24b-2 genome, among the lowest so far for basidiomycete fungi (Castanera *et al.* 2017). However, reported TE contents are highly variable (ranging between 0.1% and 42%), possibly also reflecting in part differences in sequence assembly and TE annotation protocols (Castanera *et al.* 2017).

### Circular mitochondrial genome

This study provides the first complete and circular mitochondrial genome for *D. hungarica*. Organization of the mitochondrial genome of strain PDD-24b-2 differs from that of other *D. hungarica* strains basing on previously reported physical maps (Gácser *et al.* 2002). This is not unexpected as mitochondrial genome maps differed between *D. hungarica* strains.

The mitochondrial genome of strain PDD-24b-2 is smaller (27 kb) than those of *D. changbaiensis* (35 kb; Tan and Wang 2021) and *D. cryoxerica* ANT 03-071^T^ (36 kb; L. B. Connell, personal communication) but of similar GC content (40–42%). The PDD-24b-2 mitochondrial genome contains all 15 known core protein-coding genes of mitochondria in Basidiomycetes, 23 tRNAs, and 2 rRNAs (Table 2).

**Table 2.** Comparison of the mitochondrial genomes of *D. hungarica* and *D. changbaiensis*. General statisticsMitochondrial genome contents

Content	*Dioszegia hungarica* PDD-24b-2	*Dioszegia changbaiensis* CGMCC AS 2.2309^T^
Accession no.	JAKWFO000000000	MT755637
Reference	This study	Tan and Wang (2021)
		
Size (bp)	27,226	34,853
GC content (%)	40.6	41.9
		
Protein-coding genes	*atp6*, *atp8*, *atp9*, *cob*, *cox1*, *cox2*, *cox3*, *nad1*, *nad2*, *nad3*, *nad4*, *nad4L*, *nad5*, *nad6*, *rps3*	*atp6*, *atp8*, *atp9*, *cob*, *cox1*, *cox2*, *cox3*, *nad1*, *nad2*, *nad3*, *nad4*, *nad4L*, *nad5*, *nad6*, *rps3*
tRNA	*trnA(ugc)*, *trnD(guc)*, *trnE(uuc)*, *trnF(gaa)*, *trnG(ucc)*, *trnH(gug)*, *trnI(gau)*, *trnK(uuu)*, *trnL(uaa)*, *trnL(uag)*, *trnM(cau)*, *trnM(cau)*, *trnN(guu)*, *trnP(ugg)*, *trnQ(uug)*, *trnR(ucg)*, *trnR(ucu)*, *trnS(gcu)*, *trnS(uga)*, *trnT(ugu)*, *trnV(uac)*, *trnW(cca)*, *trnY(gua)*	*trnA(ugc)*, *trnD(guc)*, *trnE(uuc)*, *trnF(gaa)*, *trnG(ucc)*, *trnH(gug)*, *trnI(gau)*, *trnK(uuu)*, *trnL(uaa)*, *trnL(uag)*, *trnM(cau)*, *trnM(cau) trnN(guu)*, *trnP(ugg)*, *trnQ(uug)*, *trnR(ucg)*, *trnS(gcu)*, *trnS(uga)*, *trnT(ugu)*, *trnV(uac)*, *trnW(cca)*, *trnY(gua)*
rRNA	*rns*, *rnl*	*rns*, *rnl*
Arg codons in mitochondrial CDS	aga: 0, agg: 1, cga: 72,	aga: 1, agg: 0, cga: 66,
	cgc: 0, cgg: 10, cgu: 16	cgc : 1, cgg: 8, cgu: 20

The additional tRNA gene in *D. hungarica* is underlined.

One major difference between the mitochondrial genome of *D. hungarica* and that of *D. changbaiensis*, the only other *Dioszegia* annotated mitochondrial genome to date, is the presence in *D. hungarica* of an additional tRNA gene, *trnR(ucu)* for arginine (Table 2). Although similar arginine codon usages are found in both strains, this is not the case for the aga and agg codons that are exclusively found in one of the mitochondrial genome. It is possible that this additional tRNA-Arg(ucu) in *D. hungarica* is used to translate the agg codon (Agris *et al.* 2007). On the other hand, in the absence of tRNA-Arg(ucu) the translation of the aga codon in *D. changbaiensis* remains unexplained.

A noticeable similarity between the mitochondrial genome of *D. hungarica* and that of *D. changbaiensis* is a high cgu codon usage for arginine (16.2% of arginine codons for *D. hungarica* and 20.8% for *D. changbaiensis*) (Table 2). Thus, as this cgu codon cannot be canonically translated by either tRNA-Arg(ucg) or tRNA-Arg(ucu) without post-transcriptional modifications (Phizicky and Hopper 2010), experiments are needed to identify yet unknown modification processes and their roles in translation in *D. hungarica* and *D. changbaiensis* mitochondria.

### Phylogenetic analysis and environmental distribution

**Fig. 3.**
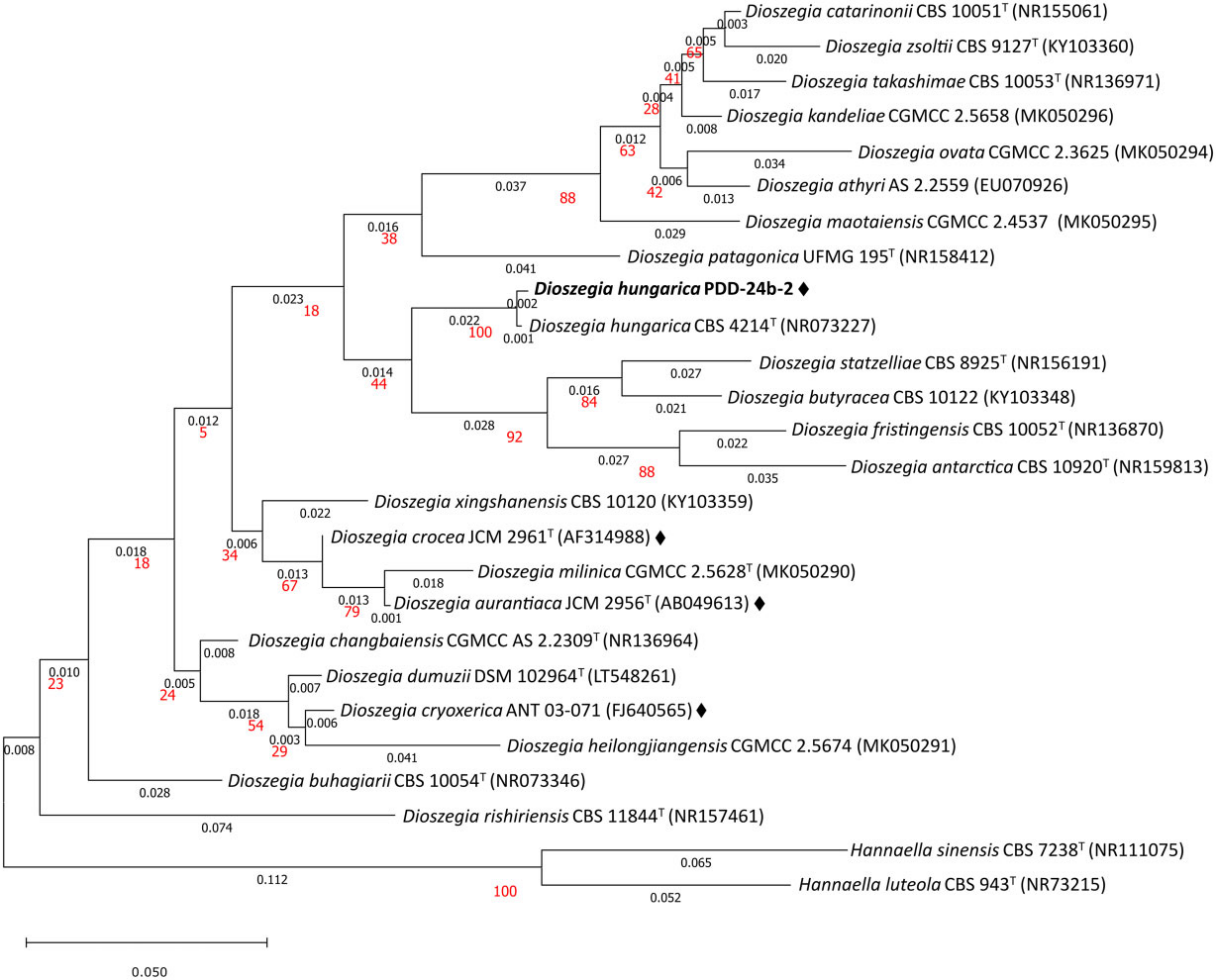
Phylogenetic analysis of the *Dioszegia* genus based on the ITS region. The tree was obtained from a sequence alignment of 462 nt of the ITS region with the Maximum Likelihood method and General Time Reversible model. Branch lengths (number of substitutions per site) are indicated under each branch, bootstrap support values (percentage of replicate trees in which the associated taxa clustered together in the bootstrap test 500 replicates) are indicated in larger fontsize. Sequence accession numbers are indicated in brackets. Diamonds (◆) indicate strains for which a draft genome is available. *Hannaella sinensis* and *Hannaella luteola* were used as outgroups.

A phylogenetic tree based on the analysis of the ITS region was constructed for 24 strains of the genus *Dioszegia*, including strain PDD-24b-2, with 2 strains of the genus *Hannaella*, as outgroups, using the Maximum Likelihood method (ITS sequence information in Supplementary Table 2). In this tree, *D. hungarica* PDD-24b-2 and the *D. hungarica* type strain are clustered together and distinct from genome-sequenced strains of other *Dioszegia* species (Fig. 3), in accordance with previous taxonomical studies (Trochine *et al.* 2017; Li *et al.* 2020).

**Fig. 4.**
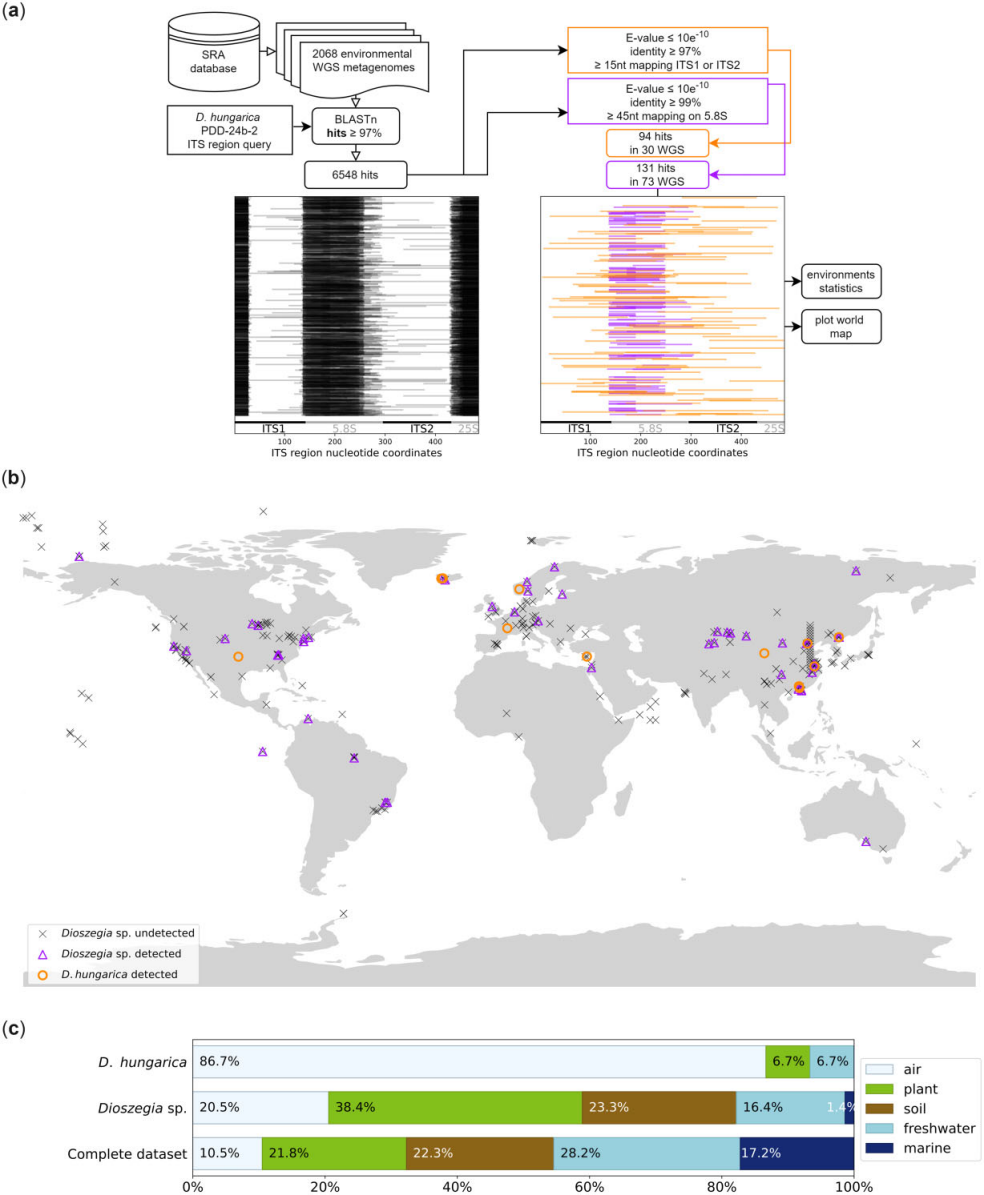
Geographical and environmental distribution of the *Dioszegia genus* and *D. hungarica* species. a) Environmental metagenome exploration pipeline. The megablast hits filtering process is indicated with unfiltered hits (left), filtered hits representing *D. hungarica* (right, light hits), or *Dioszegia* sp. sequences (right, dark hits) mapped on *D. hungarica* PDD-24b-2 ITS region. b) Geographical distribution of sequences assigned to *D hungarica* or *Dioszegia* sp. in environmental metagenomic datasets (see Supplementary Table 3 for detailed information). c) Environmental distribution of *D hungarica* and *Dioszegia* sp. compared with that of the complete metagenomic dataset.

Geographical distribution and potential habitat specificity *of D. hungarica* were investigated with a large set of public metagenomes selected to represent a wide diversity of environments, using the ITS region of strain PDD-24b-2 as a query (Fig. 4a). *Dioszegia hungarica* was detected at diverse latitudes around the world (Fig. 4b), and mostly in aerial biomes. In contrast, representatives of the *Dioszegia* genus were found to be more diversely distributed (Fig. 4c). Strikingly, ITS sequences specific of *D. hungarica* were not detected in marine samples in our dataset of selected metagenomes, nor in the Mar and TARA Ocean Gene Atlas databases (no fungal hits with over 97% identity were found). This suggests that *D. hungarica* is scarce in open sea environments. On the other hand, *D. hungarica* sequences were not detected in soil metagenomes either (Fig. 4c). This was surprising since the *Dioszegia hungarica* type strain was isolated from soil (Takashima *et al.* 2001). However, when using the GlobalFungi database, which is a terrestrial soil-focused database, ITS sequences of *D. hungarica* were detected in soil samples. Considering the significant differences in types of sequences between metagenomes (WGS, short raw reads) and the GlobalFungi database (targeted amplification of longer ITS sequences), the stringency of search parameters used in our analysis may contribute to explain this discrepancy, especially in environments with low abundance of *D. hungarica* communities. Nevertheless, the low occurrence of *D. hungarica* in oceans is somewhat paradoxical considering that strain PDD-24b-2 was isolated from a cloud of oceanic origin (Supplementary Fig. 1). We thus hypothesize that *D. hungarica* was picked up during air mass travel across France through the puy de Dôme sampling site. As such, the detection of *D. hungarica* in cloud water could serve as an indicator of air mass contact with terrestrial surfaces in future studies where detailed characterization of investigated cloud microbiomes is of interest.

In conclusion, the obtained high-quality assembled and annotated genome of the orange-pigmented psychrotrophic yeast *D. hungarica* PDD-24b-2, a major representative of the cloud microbiome, now provides a blueprint for future functional genomics analyses of this environmentally relevant fungus. This will help characterize its mechanisms of resistance to UV radiation (Inacio *et al.* 2005) and of survival in cold environments (Dalluge *et al.* 2019), contribute to develop yeast enzymatic processes at low temperatures (Vaz *et al.* 2011), and help to identify and characterize the biotic factors that play a role in cloud chemistry.

## Supplementary Material

jkac282_Supplementary_Figure_S1

jkac282_Supplementary_Figure_S2

jkac282_Supplementary_File_S1

jkac282_Supplementary_Table_S1

jkac282_Supplementary_Table_S2

jkac282_Supplementary_Table_S3

## Data Availability

The *Dioszegia hungarica* PDD-24b-2 Whole Genome Shotgun project was deposited at DDBJ/ENA/GenBank under accession number JAKWFO000000000. The genome version used in this report is JAKWFO010000000. The raw Illumina and PacBio reads were deposited at the Sequence Read Archive under accessions numbers SRR18177991 and SRR18177990, respectively. Details on genome assembly and gene model properties are provided on the MycoCosm genome portal (https://mycocosm.jgi.doe.gov/Diohu1). Strain *Dioszegia hungarica* PDD-24b-2 is available upon request to Dr Pierre Amato or Dr Françoise Bringel. Representative sequences of putative *D. hungarica* PDD-24b-2 TE families are in Supplementary File 1. Putative TEs detected in *D. hungarica* PDD-24b-2 are in Supplementary Table 1. ITS sequences used to construct the phylogenetic tree of the *Dioszegia* genus are in Supplementary Table 2. Environmental samples in which *D. hungarica* and *Dioszegia* species were searched are provided in Supplementary Table 3. The air mass trajectory of the cloud from which *D. hungarica* strain PDD-24b-2 was isolated is shown in Supplementary Fig. 1. A close-up of the Illumina read depth coverage of *D. hungarica* PDD-24b-2 rRNA gene region is provided in Supplementary Fig. 2. The homolog search scripts, environmental metagenome dataset, as well as more information on biogeographic analyses and TE mining are available at https://github.com/JarrigeD/Dioszegia_hungarica_sequencing. Supplemental material is available at G3 online.
