# Supplementary material for: High-quality genome of the basidiomycete yeast Dioszegia hungarica PDD-24b-2 isolated from cloud water
Source: G3 (Bethesda). 2022 Oct 19;12(12):jkac282. doi: 10.1093/g3journal/jkac282 (PMC9713403; doi:10.1093/g3journal/jkac282)
Supplement: jkac282_Supplementary_Figure_S1 [file jkac282_supplementary_figure_s1.docx]

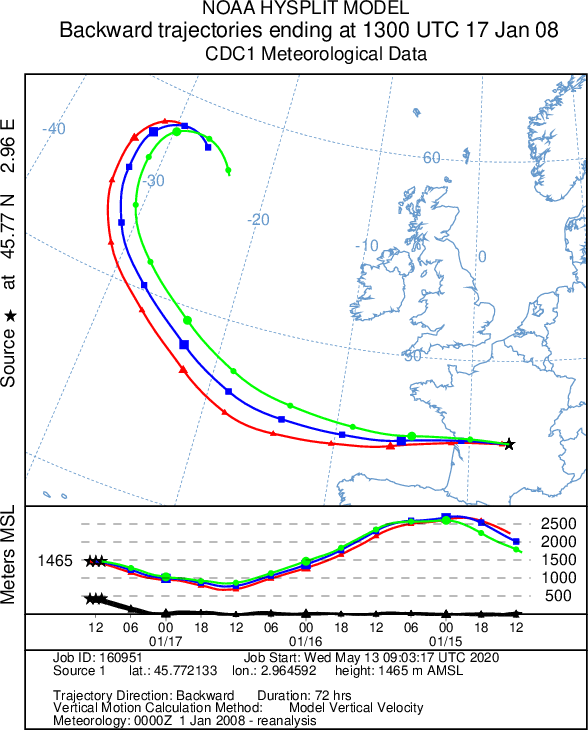


**Fig. S1. Air mass trajectory of the cloud from which D. hungarica strain PDD-24b-2 was isolated.** Top: 72 hours modelled backward air trajectories (NOAA HYSPLIT model, Stein et al. 2015 <https://doi.org/10.1175/BAMS-D-14-00110.1>) of three air masses with 1h time interval (green circles, blue squares and red triangles, respectively) over the duration of the cloud water sampling at puy de Dôme. Bottom: air mass altitude in meters above sea level (MSL). Trajectory endpoints corresponding to the time of collection of cloud water from the air masses at the puy de Dôme sampling station are indicated by black stars.
