# Supplementary material for: High-quality genome of the basidiomycete yeast Dioszegia hungarica PDD-24b-2 isolated from cloud water
Source: G3 (Bethesda). 2022 Oct 19;12(12):jkac282. doi: 10.1093/g3journal/jkac282 (PMC9713403; doi:10.1093/g3journal/jkac282)
Supplement: jkac282_Supplementary_Figure_S2 [file jkac282_supplementary_figure_s2.docx]

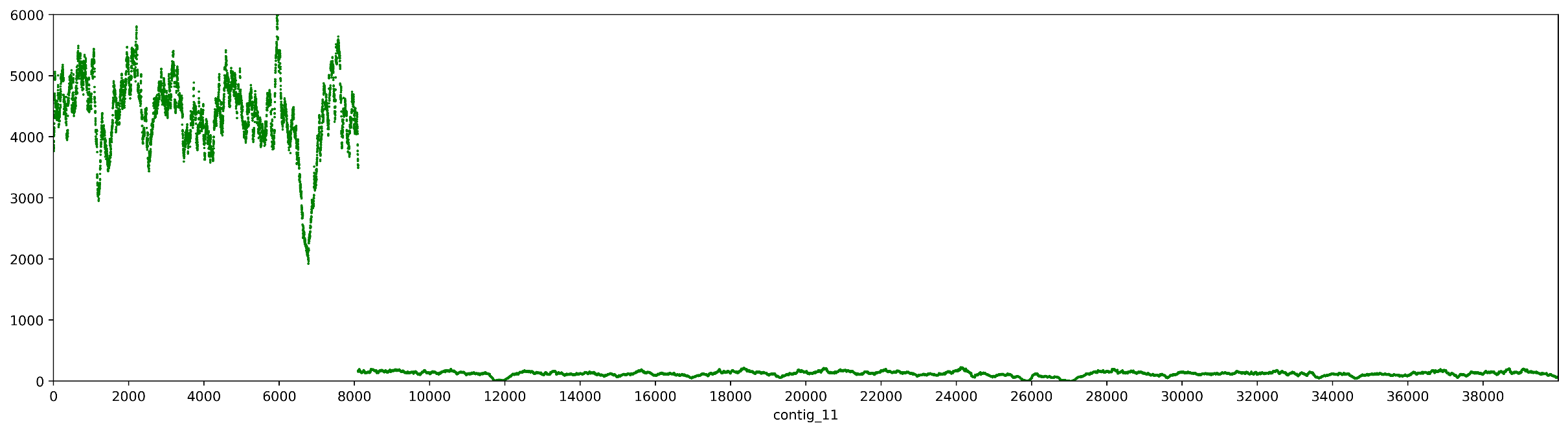

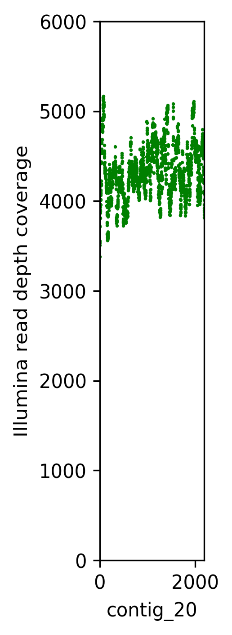


**Fig. S2. Close-up of the Illumina read depth coverage of D. hungarica PDD-24b-2 rRNA gene region.** Illumina read depth coverage on contig_20 and the first 40kbp of contig_11, plotted in python with Matplotlib.
